# Design of Planar Array Transducers Based on Connected 1–3 Piezoelectric Composites

**DOI:** 10.3390/mi12111417

**Published:** 2021-11-18

**Authors:** Jiacheng Wang, Chao Zhong, Shaohua Hao, Ning Lv, Likun Wang

**Affiliations:** 1 Beijing Key Laboratory for Sensor, Beijing Information Science & Technology University, Beijing 100101, China; 2019020178@bistu.edu.cn (J.W.); 20192289@bistu.edu.cn (C.Z.); 2019020614@bistu.edu.cn (N.L.); 2 School of Electronic Engineering, Beijing University of Posts and Telecommunications, Beijing 100876, China; haoshaohua@bupt.edu.cn

**Keywords:** 1–3 piezoelectric composite, array transducer, preparation process, transmitting voltage response, transmission property

## Abstract

To improve the transmission performance and preparation of a transducer array, two planar array transducers based on connected 1–3 piezoelectric composites as a matrix were designed. Each transducer contained 25 array elements with a gap of 1 mm between them. The length, width and height of each array element were 1 mm, 26 mm and 5 mm, respectively. Two kinds of array transducers were tested through finite element simulation and experiments. The array transducer prototype was fabricated based on two kinds of composite materials, and the fabrication cycle was short. Our results show that the maximum transmission voltage response of the two-phase 1–3 full array driver is up to 179 dB at 200–400 kHz and the acoustic radiation intensity can be increased by up to 22% compared with the traditional splicing transducer array. It is suitable for short-range target positioning and measurement. Moreover, in the single element mode, the beam of the three-phase 1–3 transducer has no sidelobe and a single element −3 dB beam width of up to 91°. Furthermore, the beam width of the two-phase 1–3 type is 54°, and the acoustic radiation breadth is improved by 40.6%. The three-phase 1–3 type array transducer has the characteristics of concentrated acoustic transmission energy of the whole array, and its −3 dB beam width is 3.5°. The beam width decreased by 12.5%, indicating that the three-phase 1–3 type transducer is suitable for short-range target detection and perception. The two array transducers have their own advantages in transmitting the voltage response and beam width, which must be selected on the basis of the requirements of practical applications.

## 1. Introduction

Ultrasonic transducers are widely used in underwater acoustics [1] and exist in the form of arrays comprising multiple transducer elements. Using transducers in this way can improve the precision and accuracy of underwater acoustic detection [2]. Transducer array technology was initially applied by the military [3,4]. For example, acoustic homing torpedoes realize electroacoustic signal conversion through a torpedo head transducer array and guide the torpedo toward its target. Moreover, underwater acoustic fuse technology can detonate mines by receiving acoustic waves from the target.

Generally, transducer array technology has been widely used in underwater detection [4], nondestructive testing [5,6], and other fields. Based on different structures, transducer arrays can be divided into linear, area, ring, etc. The American company MICRO STAR INTERNATIONAL (MSI) [7] designed an underwater acoustic arc transducer array. The element diameter of the array was 100 mm, and the beam opening angle could reach 150°, but this transducer’s working bandwidth was extremely low, only roughly 10 kHz. Li et al. [8] designed a cylindrical transducer array on the basis of 1–3-2 piezoelectric composites; its transmitting voltage response could reach 139 dB, and this type of transducer has a 360° directivity in the horizontal direction. However, due to the spacing of active materials in the cylindrical array, the acoustic radiation surface of each element was limited, and the transmitting voltage response was relatively small. Teng [9] prepared 12 Tonpilz transducer array elements using piezoelectric ceramics and embedded them into the cylindrical array structure through a splicing method to form a cylindrical underwater acoustic transducer array. The overall transmitting voltage response of the array was up to 153.96 dB, but the fabrication of the array and the assembly of each element were highly complex, and hence the yield of the transducer array was extremely low. Lv et al. [10] designed a multi-element piezoelectric composite transducer through a “dice-and-fill” method. Its maximum transmitting voltage response is 151.3 dB. In addition, adding a matching layer to the active material expanded the bandwidth of the transducer; Zhang et al. [11] prepared a new capacitive micromechanical ultrasonic transducer array and discovered that a 1 × 16 linear array had good directivity and propagation distance at 400 kHz.

However, the current underwater acoustic transducer array is usually made by manually splicing multiple transducer elements, and its overall transmitting voltage response is 140–150 dB. Moreover, the construction process for the transducer array is complex, and fabricating the transducer array takes a long time. Meanwhile, in the transducer array, the consistency of elements can improve the acoustic radiation performance [9]. Therefore, to ensure the consistency of each transducer element, the production environment and the conditions of each transducer must be strictly identical. The complex array splicing process, the long array preparation cycle and the consistency of the high-precision transducer elements complicate the manufacturing process of the transducer array and significantly reduce its yield. Therefore, improving the forming process of the transducer array, reducing the preparation cycle of the transducer, forming the difficulty of the array, and ensuring the consistency of the transducer elements are the keys to address existing problems [12].

1–3 piezoelectric composites are the most used active material in underwater acoustic transducers, which not only retains the inherent advantages of the original piezoelectric ceramics [13] but also greatly improves the overall performance of piezoelectric materials [13,14]. Meanwhile, 1–3 piezoelectric composites are mostly prepared through a “dice-and-fill” method [8,15]. The preparation is relatively simple and is not time-consuming. Therefore, they have good application prospects and practicability in the field of sensitive materials of transducers. Walter et al. [16] studied the dicing speed and defect degree of PZT ceramics using 1–3 connected composite materials at 5 MHz. In contrast, when ceramic A is cut at a speed of 12 mm/s, its defect rate can be less than 1%. Finally, an SnBi solder with a low melting temperature of 138 °C was used for hot rod welding of materials and circuit boards. Herzog et al. [17] prepared 1–3 piezoelectric composites using PMN-PT and epoxy by the “dice-and-fill” method. The electromechanical coupling coefficient is up to 0.61, which is 27.9% higher than that of PZT. In addition, the bandwidth of the ultrasonic probe made of this composite material can reach 88%, which is higher than that of a PZT transducer. It brings research value to a new type of ultrasonic transducer probe, that may have high sensitivity and high bandwidth in the future. Rodrigues et al. [18] designed a 2-D phased array ultrasonic transducer based on the 1–3 piezoelectric composite. This research improved the energy transmission between the transducer and the receptor, optimizing the echo responses, which is promising for submarine applications. Zhang [19] researched ultrasonic linear phased arrays both using COMSOL and experimentally. A high-frequency, ZnO-based linear array transducer was fabricated with a combination of silicon processing and piezoelectric thick film deposition, showing that the growth of ZnO film was effectively inhibited.

In this paper, to improve the transmitting performance of the transducer array and its preparation process, 1–3 piezoelectric composites were used as the active material matrix, the piezoelectric rods were separated into uniform composite samples through “dice-and-fill” technology, and the piezoelectric ceramic array was then formed. It was divided into multiple active material elements by preparing electrodes using a mask. Finally, the water-tight layer was sealed to form a 1–3 composite planar array transducer to compensate for the shortcomings of the current array. At this time, the transducer array was formed by the parallel splicing of multiple piezoelectric composites, which greatly reduced the preparation cycle and difficulty of manually splicing the transducer array. In addition, the array electrode can separate each array element to form a transducer array structure with array elements arranged. Given that the integrated preparation process of 1–3 piezoelectric composites can ensure the consistency of transducer array elements, it has great application prospects in the future of underwater acoustic detection and marine imaging.

## 2. Design of a 1–3 Piezoelectric Composite Array Transducer

A 1–3 piezoelectric composite typically comprises 1D linear piezoelectric ceramic rods and 3D perfusion polymers [16,20]. In this paper, two kinds of 1–3 piezoelectric composites were selected to fabricate a planar array transducer and the transmitting performance of the transducer array formed by these two kinds of composite material was studied. The first is a two-phase 1–3 piezoelectric composite composed of piezoelectric rods and a rigid polymer (epoxy resin is usually used). This vibrator has greater rigidity, and the mechanical clamping effect of rigid polymers grants the vibrator stronger stability, which is a widely used active material for underwater transducers. The other is a 1–3 piezoelectric composite comprising piezoelectric rods, a rigid polymer, and a flexible polymer, which is a new type of composite-phase piezoelectric composite [21]. The flexible phase embedded in the active material can reduce the mechanical binding on both sides of the composite array. This structure promotes the vibration of the piezoelectric phase and improves the electromechanical coupling coefficient, the characteristic impedance and the transverse coupling vibration of the 1–3 piezoelectric composite. When the rigid 1–3 piezoelectric composite is used as the transducer, the transducer can have a large acoustic transmission intensity because of its high hardness. On the other hand, for the three-phase 1–3 piezoelectric composites, the planar coupling of each element can be reduced due to the addition of flexible polymers on both sides of each element. This decoupling allows the transducer to operate with a reduced beam sidelobe formation.

**Figure 1:**
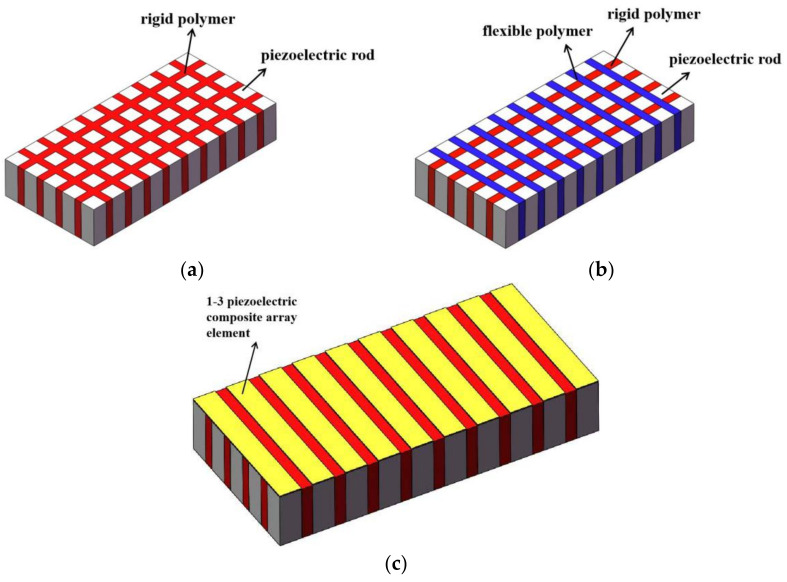
Two types of 1–3 piezoelectric composite and array structure. (**a**) Two-phase of 1–3 piezoelectric composite; (**b**) Three-phase of 1–3 piezoelectric composite; (**c**) Structure of 1–3 piezoelectric composite array.

Each of these two piezoelectric composites is vertically listed as a driving element, and the array structure is shown in Figure 1. The two types of 1–3 piezoelectric composite arrays were used as sensitive components and encapsulated by a watertight layer so that the whole composite array formed an underwater transducer array. According to the existing casting mold and metal plate size, we set the length, width and height of the material to 76 mm, 26 mm and 5 mm, respectively. The length and width of the composite array are 1 mm and 26 mm, respectively, and the element-to-element spacing is 1 mm. The elements in the array can be driven and controlled on the basis of our requirements. Thus, the fabrication process of the transducer array is optimized. This structure has not been reported in previous literature.

**Figure 2:**
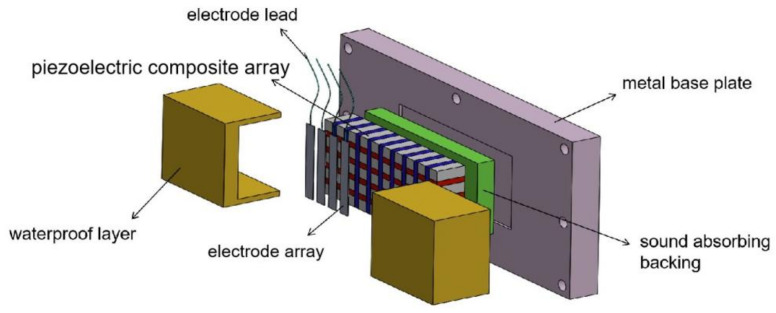
Structural design of the 1–3 piezoelectric composite planar array transducer.

Taking the three-phase 1–3 piezoelectric composite as an example, the structure of the planar array transducer designed in this paper is shown in Figure 2. The planar array transducer comprises a metal base plate, a sound absorption backing, a piezoelectric composite array, an electrode array, leads, and a watertight layer. The elements made of piezoelectric composites were arranged longitudinally, and the electrodes were divided and fabricated through a mask method. Then, the transducer array could be used after encapsulation by the waterproof layer.

## 3. Underwater Simulation on the Two Types of 1–3 Piezoelectric Composite Arrays

Before fabricating the array transducer, a feasibility analysis on the designed transducer through a simulation must be conducted, which is also of significance for the finite element analysis (FEA). In this experiment, the FEA software ANSYS 15.0 was used for simulation. The FEA aims to construct the overall simulation model and then divide the model into multiple element structures by dividing the grid. Finally, all elements are solved and summed by the solver.

The core part of the transducer is the piezoelectric vibration component. To facilitate the calculation, the structure of other parts inside the transducer is ignored. The simulation results of the sensitive components can be used to study the transmitting performance of two 1–3-type array transducers. When modeling the water field, the entire water field is usually divided into three areas, namely, the near field, the far field and the absorption boundary. The whole effect of the transmitting acoustic wave from the near field to absorbing the boundary acoustic wave from a long distance can be accurately simulated by the meshing finite element method. Based on the acoustic conditions, the active material must be completely wrapped in near-field water. Far-field water must be confined to acoustic far-field conditions [22], as shown in Equations (1) and (2):

(1) $$r\geq n\frac{D^{2}f_{a}}{v}$$

(2) $$r\geq n\frac{v}{f_{b}}$$

where *r* is the radius of the water field, *D* is the maximum radiation surface of the transducer (roughly 14 mm), *v* is the sound velocity in water (1500 m/s is taken), *n* is the amplification factor (ranging from 3 to 5), and *f_a_* and *f_b_* are the upper and lower limits of the analysis frequency, respectively. For the array transducer in this paper, the transmitting frequency was set to 200–400 kHz. The value range of the boundary water can be obtained by substituting Equations (1) and (2). Then, *r* = 200 mm can be obtained.

**Figure 3:**
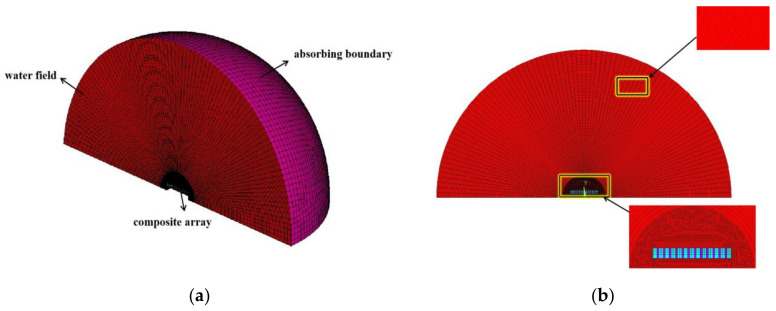
Water field simulation modeling in ANSYS. (**a**) A three-dimensional diagram of the water field; (**b**) Local structure diagram of the water field.

In the pre-processing, the 1–3 piezoelectric composite model was set to 38 mm in length, 13 mm in width and 5 mm in thickness. PZT-5A was used for the piezoelectric phase in this experiment. A total of 618 epoxy resins and 704 silicone rubbers were used for the rigid polymer and flexible polymer. The polarized piezoelectric ceramic has anisotropic properties, while the two polymers are isotropic. Specific material parameters are shown in Table 1 [23,24,25]. Following this, the material distribution, composite array, water field, and absorbing boundary had to be divided into grids. Finally, the model was swept with the “SWEEP” function of ANSYS, and the 3D finite element model of the water field could then be obtained, as shown in Figure 3.

After the finite element model was obtained, a harmonic AC voltage of 1 V was applied to the upper and lower surfaces of the composite array as vibration excitation. Concurrently, symmetric boundary constraints and fluid–solid coupling conditions were applied to all fluid–solid contact surfaces.

### 3.1. Transmitting Voltage Response of Composite Arrays

The array transducer designed in this paper was formed by integrating 1–3 piezoelectric composites, which are characterized by the uniform arrangement of each element. Through the external excitation of voltage, the independent element in the transducer can be driven and transmitted. Therefore, the working advantages of the two types of transducers can be judged by comparing the transmitting properties of the elements of the two types of transducers. The transmitting voltage response [26] is typically used to represent the intensity of the acoustic wave transmitted by the transducer under voltage excitation, which is represented by *S*_V_, and its physical expression can be determined on the basis of Equation (3):

(3) $$S_{V}=\frac{p_{0}d_{0}}{u_{e}}$$

where *d*_0_ represents the distance from the observation point to the sound center of the transducer, *p*_0_ is the sound pressure in the water at the observation point, and ue is the input voltage of the transducer (unit = Pa·m/V). For the transmitting transducer, a larger transmitting voltage response can deliver a stronger acoustic radiation effect. In general, researchers often use the transmitting voltage response level *S*_VL_ to represent the transmission performance of the transducer, which is obtained by taking the logarithm of the ratio of the transmitting voltage response value to the reference value. The unit is dB. The reference value is usually 1 μPa·m/V, and its mathematical expression is given by Equation (4):

(4) $$S_{VL}=20\mathrm{lg}\frac{|p_{0}|d_{0}}{\left|u_{e}\right|}+120$$

After modeling and solving the calculation in water, the sound pressure is extracted using the pick-point function of time course post-processing (POST 26). The sound pressure value is added into Equation (4), and the simulation curves of the transmitting voltage response of two kinds of transducers can be obtained.

#### 3.1.1. Single Element Transmitting Performance of the Array Transducer

**Figure 4:**
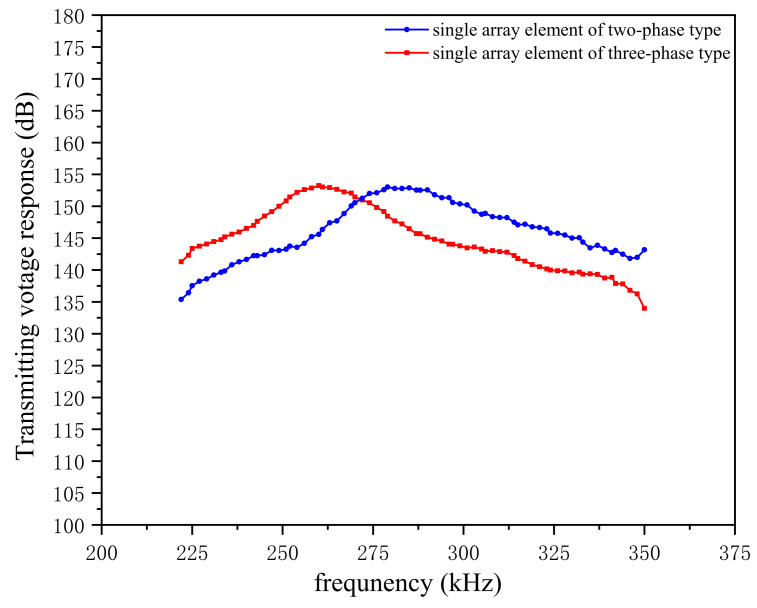
Transmitting voltage response curves of single elements of the two 1–3 piezoelectric composite by simulation.

An element at the center of the sensitive material was taken, and an AC voltage signal with an amplitude of 1v was applied on its upper and lower surfaces. After obtaining the sound pressure in the water field, the transmitting voltage response simulation curves of the two composites can be obtained on the basis of Equation (3), as shown in Figure 4.

Figure 4 depicts the center element transmitting voltage response of the 1–3 two types of composites in water. Among them, the maximum transmitting voltage response of the two-phase type can be 153.8 dB, and the maximum transmitting voltage response of the three-phase type is 152.9 dB, which is slightly lower than that of the two-phase type. Comparing the response curves of the two materials, we find that the fluctuation degree of the transmitting voltage response of the two-phase array element is slightly higher than that of the three-phase type. The composite, after adding the flexible polymer, is relatively stable at work. The reason is because the flexible polymer has a decoupling effect [21], which is conducive to the concentration of vibration energy in the thickness of the vibrator.

#### 3.1.2. Full Array Performance of the Array Transducer

**Figure 5:**
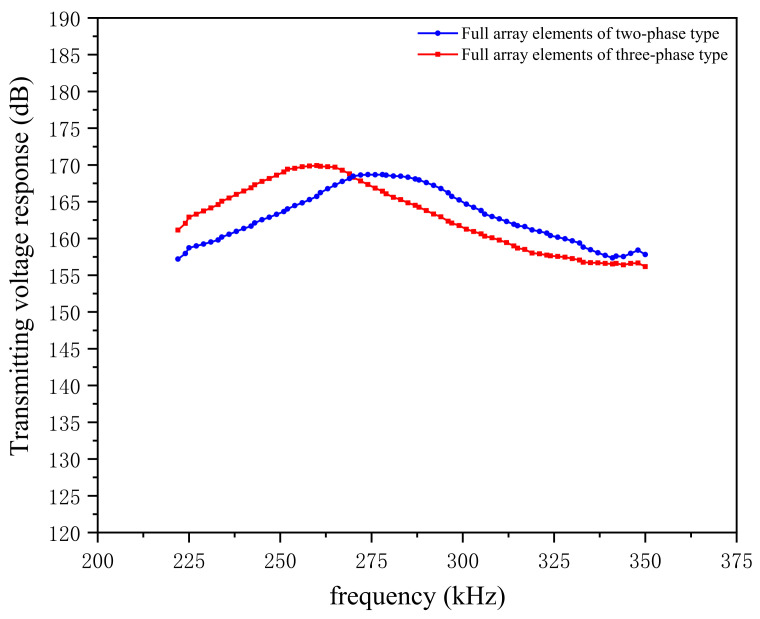
Transmitting voltage response simulation curves of all elements in two types of 1–3 piezoelectric composite array.

If AC voltage excitation is applied to all array elements, the transducer will be in the working mode of all the elements of the array. For the transducer array, the array must achieve single-element and overall transmissions of the whole element. Through this, the elements’ selectivity of the transducer array conforms to controllable requirements. Applying an AC voltage with an amplitude of 1 V to the upper and lower surfaces of all the elements in the two types of 1–3 composites, the transmitting voltage response curves of the array in the water can be obtained by the method described in the previous section. The transmitting voltage response simulation curves of two types of 1–3 piezoelectric composites in full array are shown in Figure 5.

Figure 5 exhibits the transmitting voltage response simulation of the two composites in the whole array. The intensity of the transmitting voltage response is closely related to the number of connected drive elements. The volume fraction of piezoelectric materials significantly impacts the transducer response. When the volume fraction of the ceramic phase increases, the vibration function of the system improves accordingly, and the intensity of the sound wave also strengthens. Moreover, when the two composites are near the resonance point, the two-phase type composite can reach a maximum of 168.7 dB. Meanwhile, the three-phase type can be increased to 169.9 dB, indicating that in an ideal state the two array transducers have a similar degree of acoustic transmission intensity when all the elements are driven.

Using ANSYS’s water field FEA method, a feasibility analysis of the two types of 1–3 composite array was conducted. The array transducer designed with two types of 1–3 structures can achieve a greater acoustic transmission intensity, and the maximum transmitting voltage response when driven by the full beam can be up to 169.9 dB, which is improved compared with the traditional splicing transducer array.

## 4. Fabrication of the 1–3 Piezoelectric Composite Arrays and the Transducer Prototype

### 4.1. Fabrication of the Two 1–3 Piezoelectric Composite Arrays

**Figure 6:**
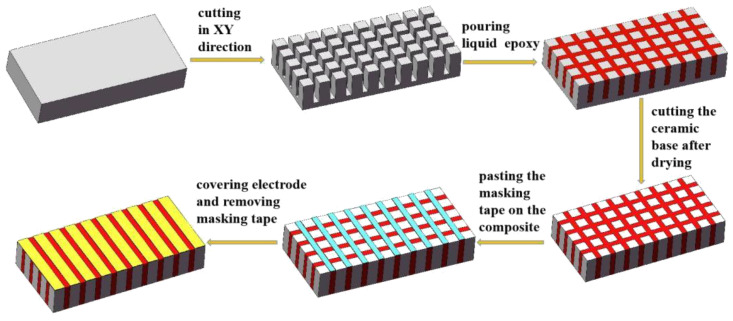
Fabrication method of the two-phase 1–3 piezoelectric composite array.

**Figure 7:**
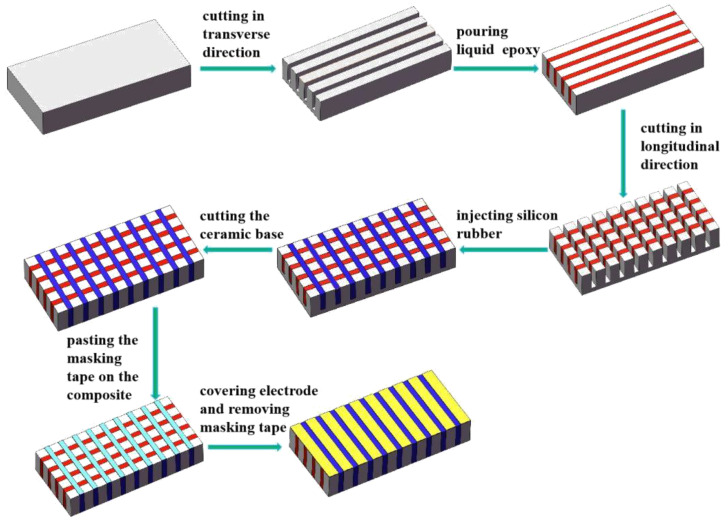
Fabrication method of the three-phase 1–3 piezoelectric composite array.

On the basis of the piezoelectric composite array model and the material size designed in the finite element analysis, two planar array transducers were fabricated. A two-phase 1–3 piezoelectric composite array and a three-phase 1–3 piezoelectric composite array were fabricated on the basis of the “dice-and-fill” technology [14,27,28,29]. In this experiment, the method of mask preparation was added to separate each element independently. The length, width and thickness of the two material samples were 76 mm, 26 mm and 5 mm, respectively, and the volume fraction of the piezoelectric phase was 45.5%. At the same time, the length, width and thickness of each composite element were 1 mm, 26 mm and 5 mm, respectively. The spacing between the elements was 1 mm, and the number of elements was 25. The specific preparation method is shown in Figure 6. For the three-phase 1–3 type, the XY direction is cut, and then the epoxy resin and silicone rubber are poured in. The three-phase 1–3 composite array was prepared in accordance with Figure 7.

**Figure 8:**
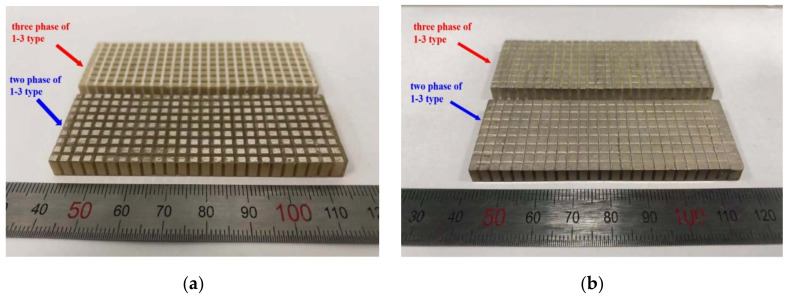
1–3 piezoelectric composite and its array structure. (**a**) Two samples of 1–3 piezoelectric composite materials; (**b**) 1–3 piezoelectric composite array after electrode preparation by mask.

After the material was prepared using the method shown in Figure 6 and Figure 7, two types of 1–3 piezoelectric composite were obtained, and the arrays of the sample and electrode after preparation are shown in Figure 8.

### 4.2. Fabrication of the Planar Array Transducer Prototype

**Figure 9:**
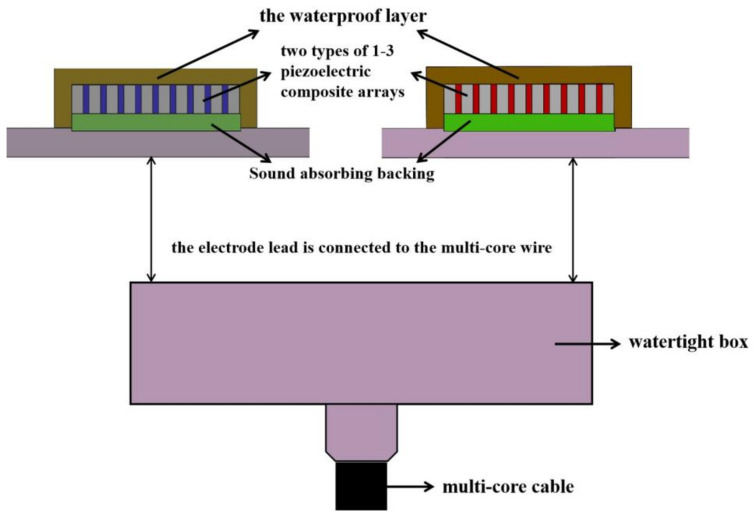
Section structure of the planar array transducer.

The prototype of the planar array transducer is composed of functional components and a waterproof metal water tank. First, the transducer comprises functional components of a metal base plate, sound-absorbing backing, composite array, and waterproof layer [30], which are also the core components of the transducer. Second, the back of the metal base plate comprises a waterproof metal water tank and multi-core cables. The specific sectional view of the transducer is shown in Figure 9. The piezoelectric composite and the sound absorption backing line were fixed on the back of the metal plate after the bonding to form a stable structure. The electrode lead was connected to the multi-core cable. After applying voltage, the piezoelectric sensitive materials could produce vibrations through the inverse piezoelectric effect and transmit acoustic waves outward. The sound-absorbing backing can suppress the reverse acoustic wave, thereby concentrating the transmission area of the acoustic wave in the front. Finally, the planar array transducer can be formed by wrapping the piezoelectric composite with a waterproof layer. The driving device can be connected by the multi-core wire in the cable, thus controlling the transducer array element. The specific fabrication method is as follows:

1. The 1–3 piezoelectric composite and the sound absorption backing were firmly pasted with adhesive, fixed on the metal base plate, and then cured for 24 h at room temperature (25 °C).
2. After curing, the remaining dirt on the bottom plate was cleaned with pure ethyl alcohol, and the leads on the composite were drawn out of the metal base plate.
3. The watertight layer potting molds were scrubbed, and a layer of release agent was applied to ensure that the potting polyurethane could be removed smoothly. After smearing evenly, the sealing molds were fixed with screws on both sides of the piezoelectric composite, on the metal base plate, and baked until dry for 1 h at 60°.
4. Polyurethane was equipped at a constant temperature of 75 °C. The ratio of polyurethane to curing agent must be 10:1. Graphite was added to the liquid polyurethane and vacuumed for 3–5 min to complete the preparation.
5. The molds were removed from the drying baker, and liquid polyurethane was injected into them and then baked again at a constant temperature of 60° for 24 h.
6. The sealing mold from the metal base plate was unloaded, and the prototype of the planar array transducer was prepared.

**Figure 10:**
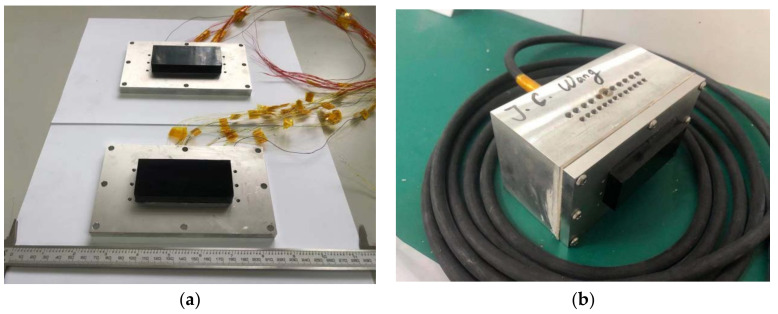
Planar array transducer prototypes. (**a**) Two types of transducer prototypes based on 1–3 piezoelectric composites; (**b**) Prototype of assembled planar array transducer before test.

Based on Figure 9, given that the planar array transducer designed in this study can realize the driving effect of multiple elements, the element selection function of the transducer can be realized only by connecting the composite array with the multi-core cable in the waterproof tank. Through this fabrication method, two kinds of 1–3 array transducer prototypes can be obtained, as shown in Figure 10, wherein the transducer with the red lead is a two-phase type and the yellow lead is a three-phase type. The method of forming the transducer array with 1–3 piezoelectric composites can greatly reduce the process cycle of the splicing array. In addition, on the condition that the sample is uniform, the electrodes were neatly divided using the mask, which gives a better consistency to the array elements.

## 5. Results

### 5.1. Effective Electromechanical Coupling Coefficient Test for the 1–3 Composite Array

The effective electromechanical coupling coefficient is a physical quantity that describes the electromechanical conversion capability of piezoelectric active materials. The larger the effective electromechanical coupling coefficient, the stronger the material’s electromechanical conversion capability. Generally, it can be obtained from the resonant frequency and anti-resonant frequency of the transducer using Equation (5) [31]:

(5) $$k_{e}=\sqrt{\frac{f_{p}^{2}-f_{s}^{2}}{f_{p}^{2}}}$$

In Equation (5), *f_s_* and *f_p_* represent the resonant frequency and anti-resonant frequency of the piezoelectric vibrator, respectively. An impedance analyzer (Agilent 4294A, Agilent Technologies, Inc., Santa Clara, CA, USA) was used to test the resonant frequencies and anti-resonant frequency of the two types of composites. Finally, we can obtain the effective electromechanical coupling coefficient of the two materials using Equation (5).

**Figure 11:**
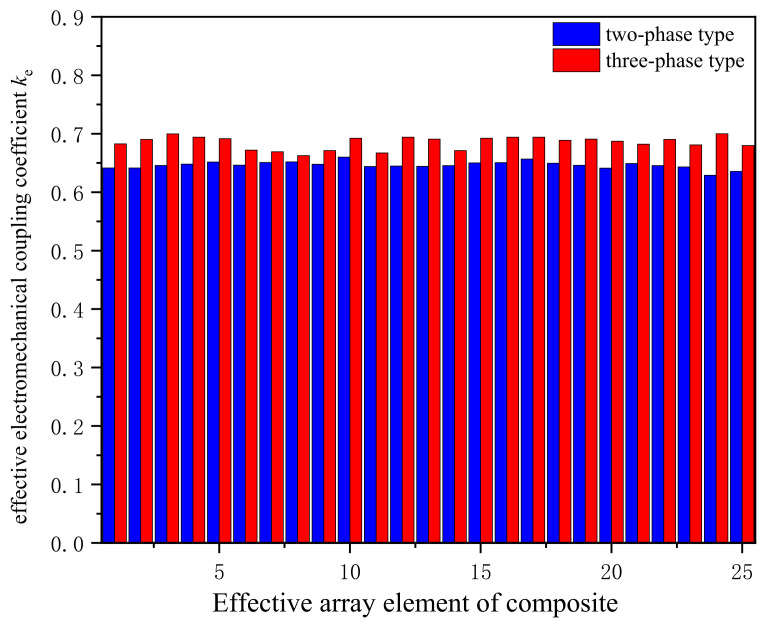
Effective electromechanical coupling coefficients of two 1–3 piezoelectric composites.

Figure 11 depicts the bar charts of the effective electromechanical coupling coefficients of two 1–3 piezoelectric composite arrays. The effective electromechanical coupling coefficient without any load condition can accurately characterize the electromechanical conversion ability of piezoelectric materials. The three-phase 1–3 piezoelectric composite is a new 1–3 connected structure active material discovered recently. Due to the decrease in the mechanical binding force on both sides of the array element, the vibration effect of each element is improved, which can concentrate the vibration energy of the material and improve the uniformity and purity of the acoustic waves transmitted by the transducers. It can be found that the effective electromechanical coupling coefficient of the three-phase 1–3 array is up to 0.70, and the effective electromechanical coupling coefficient of the three-phase 1–3 composite element is generally higher than that of the two phase 1–3 type (the maximum difference is 7.7%). Therefore, the quality of acoustic waves transmitted by the three-phase 1–3 type transducer can be improved, and the energy loss of the system will be effectively reduced.

### 5.2. Underwater Performance of the Planar Array Transducer

The two kinds of transducers were fixed in water, and the relationship between the array element and its resonant frequency could be obtained by connecting the lead of the transducer array element with the impedance analyzer.

**Figure 12:**
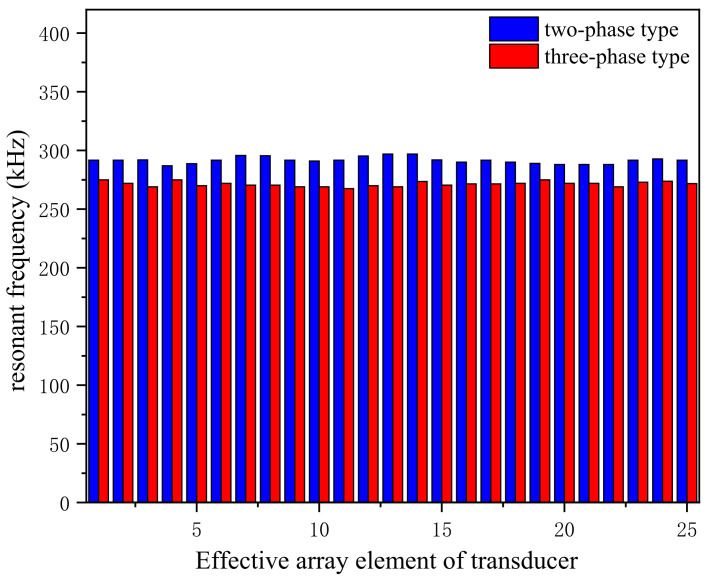
Transducer array element consistency test.

For high-frequency transducers, the resonant frequency consistency of the array elements is related to the transmitting performance of the whole array. Figure 12 depicts the relationship between the elements and resonant frequencies of the two kinds of transducers in water, where the number of effective elements is 25. The resonant frequencies of the two 1–3 transducers have a good consistency, indicating that the integrated array can cause significant similarities between each array element of the transducers. Taking the average resonant frequency of the two composite arrays, it is found that the resonant frequency of the two-phase 1–3 type is 291.64 kHz and that of the three-phase 1–3 type is 271.36 kHz. Interestingly, the resonant frequencies of the two 1–3 type composites are highly consistent. Compared with their respective average values, the resonant frequency difference between the elements is within 4.64 kHz. The water pressure will load the transducer when the transducer works in water. However, we find that the load has little effect on the resonant frequency, and the difference between each element’s resonant frequency is quite small. Comparing the resonant frequencies of the two curves, the three-phase 1–3 type has a lower resonant frequency and can reach the resonant point easier than the two-phase 1–3 type.

**Figure 13:**
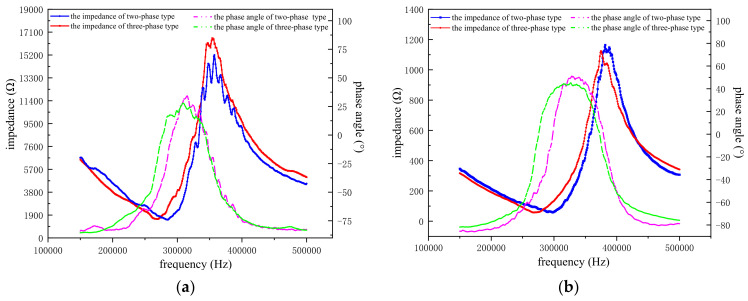
Underwater impedance-phase of single element and full array of the planar array transducer. (**a**) Two kinds of transducer single array element impedance-phase curves in water; (**b**) Two kinds of transducer full array impedance-phase curves in water.

The different active materials of the two kinds of transducer will inevitably lead to differences in the working performance of the transducer in water. The performance of two kinds of transducers in water was studied. First, the two transducer prototypes were fixed in water with metal rods. Then, the impedance analyzer was used to test the central array element and the full array elements of the two transducers in water. Finally, we obtained the impedance–phase curves of the two 1–3 array transducers in water, as shown in Figure 13. Meanwhile, the resonant frequency, anti-resonant frequency, and –3 dB bandwidth of the two array transducers can also be obtained by the impedance analyzer. We tested the prototype three times in the same environment, and the average values are presented in Table 2.

Figure 13a depicts the impedance and phase angle relationship of two transducer single elements at different frequencies. We found that the two element curves have a similar trend. However, the two-phase 1–3 material has a relatively low impedance, so the voltage generated by the active material will be higher and the intensity of the transducer’s acoustic transmission will be relatively high under the same conditions. From the perspective of vibration mode analysis, the two-phase 1–3 curve has more burrs, and its thickness vibration mode is not uniform, which is mainly caused by the clamping action of rigid epoxy resin around the piezoelectric column. Hence, the vibration of the two-phase 1–3 active material will be accompanied by a small amount of energy loss. Compared with the two-phase 1–3 type, the three-phase 1–3 type array element curve has a relatively smooth character because the flexible silicone rubber has a certain decoupling function, which can make the vibration mode of the three-phase active material pure. In addition, the −3 dB bandwidth of the three-phase 1–3 type is slightly higher than that of the two-phase 1–3 type because the flexibility of the silicone rubber promotes the vibration of the piezoelectric rods and increases the working bandwidth by increasing the mechanical loss of the material.

Figure 13b shows the impedance–phase curves of the two transducers when they are driven by full elements in water. With the increase in the number of added elements, the overall impedance values of the two transducers decreased correspondingly, and the current-carrying capacity of the system increased. When the frequency is near the resonant frequency, the two transducers have a very similar minimum impedance, which is lower in the two-phase 1–3 type than in the three-phase 1–3 type, with a minimum impedance of 55.95 Ω. A transducer system with a lower load can generate a higher voltage, which enables a higher transmission voltage response when the transducer is operating at resonance. In addition, when the number of array elements increases, the volume fraction of silicone rubber increases and the bandwidth difference between the two transducers is also significant. The maximum −3 dB bandwidth of the three-phase 1–3 transducer can be increased by 28.8%, which helps to widen the acoustic channel of the transducer, allowing the transducer to transmit more signals. Comparing the fluctuation of the two curves, the curve of the three-phase 1–3 type is still relatively smooth, and the active material mode is relatively pure, which is conducive for the transducer to transmit a relatively pure acoustic signal.

### 5.3. Transmitting Performance Test of the Transducer

**Figure 14:**
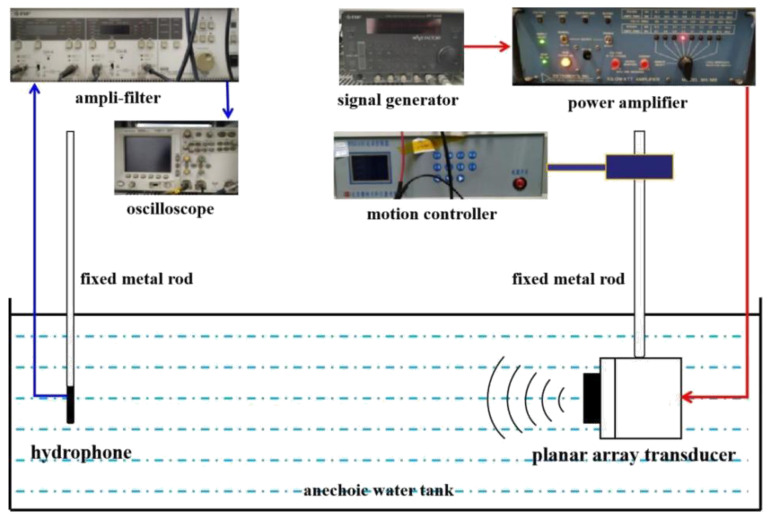
Underwater test system for the transducer.

**Figure 15:**
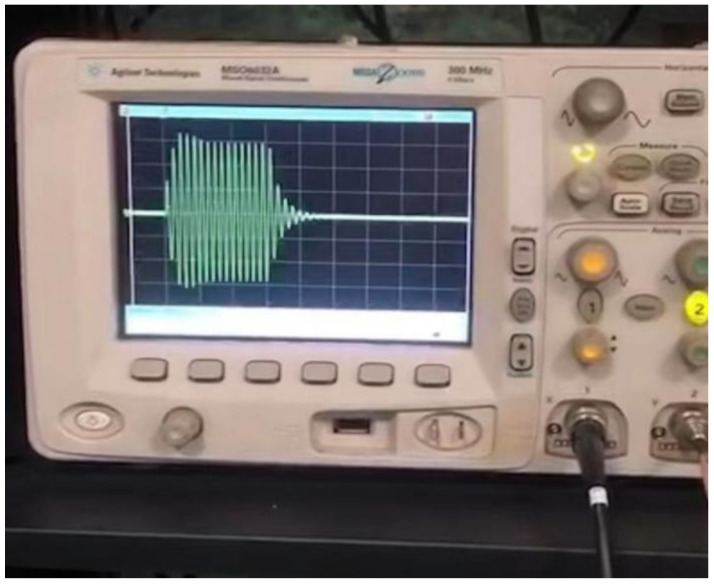
A waveform that the hydrophone received when the transducer transmitted a sound wave.

The working quality of the transducer can be evaluated by testing its underwater transmission performance. Based on the testing requirements, the array’s element lead wire should be connected to the multi-core cable, and the underwater acoustic test system can be used to test the performance of the transducer prototype in the anechoic pool [32]. The structure of the test system is shown in Figure 14. The underwater acoustic testing system comprises a transducer prototype, hydrophone (TC 4035, TELEDYNE RESON, Inc., Fabriksvangen, Slangerup, Denmark), signal generator (NF WF1946B, NF Corporation, Inc., Kohoku-ku, Yokohama, Japan), power amplifier (INSTRUMENTS M8, INSTRUMENTS, Inc., San Diego, CA, USA), amplifier filter (NF 3628, NF Corporation, Inc., Kohoku-ku, Yokohama, Japan) and motion controller (WNSC600, Winner Optical Instruments Group Company, Inc., Beijing, China). The signal waveform received by the hydrophone when the transducer transmits the sound wave is shown in Figure 15.

The transducer prototype was fixed on the rotating platform using a metal bar, and the rotating platform was connected to the motion controller. To ensure the accuracy of the acoustic wave receiving, the turntable angle was adjusted to the direct opposite of the hydrophone. The specific environment recorded for the test environment before the test is shown in Table 3.

#### 5.3.1. Testing the Transmitting Voltage Response of Two Types of Array Transducers

Connecting the lead wire of the center element and all the array elements of transducers with a multi-core cable, the transmitting voltage response of the transducers was tested. Setting the scanning frequency of the test system to 200–400 kHz, the transmitting voltage response curves of the transducer’s element and full array can be obtained.

**Figure 16:**
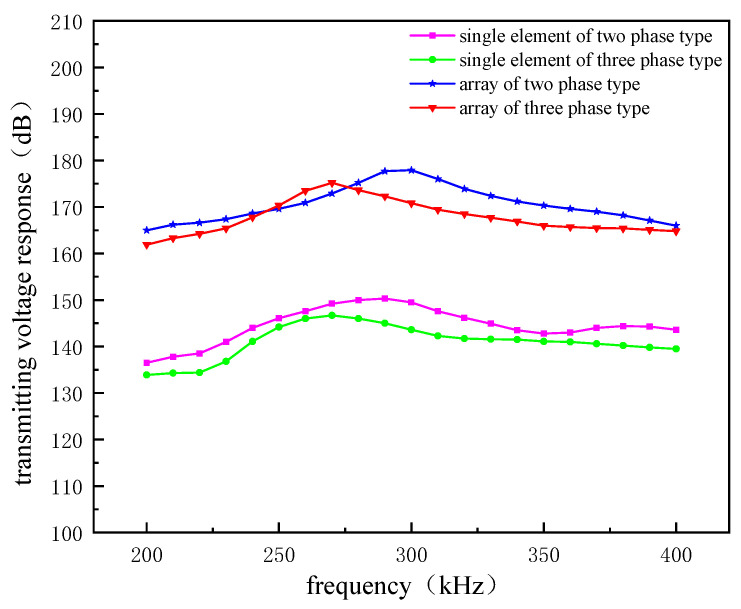
Transmitting voltage response curves of the two array transducers in single-element mode and full-array mode.

To ensure the accuracy of the test, we stimulated the external voltage of the same signal three times each for the single and full array elements of the two transducers, following which we obtained highly similar transmission voltage response curves, of which a group of transmission voltage response curves are shown in Figure 16. When the transducer is in a single-element driving mode, the transmitting voltage response of the two-phase 1–3 is above that of the three-phase 1–3, and the maximum response value is 153 dB, indicating that under the same excitation the two-phase 1–3 type’s element has a higher transmitting acoustic intensity than the three-phase 1–3 type. When the transducer is in a full array driving mode, the three-phase 1–3 type curve rises faster, and in the frequency band 250–275 kHz the transmitting voltage response amplitude exceeds that of the two-phase 1–3 type. When the two types of transducers are in resonance, the transmitting voltage response of the two-phase 1–3 type reaches 179 dB, and that of the three-phase 1–3 type reaches 175 dB. Both 1–3 arrays have a high transmitting voltage response, and the acoustic radiation intensity can be increased by up to 22% compared with the traditional splicing transducer array.

The two planar array transducers produced by the integrated process have achieved remarkable results in the transmitting voltage response. The two-phase 1–3 piezoelectric composite has extreme hardness, and the epoxy resin not only effectively separates the piezoelectric rods but also weakens the transverse coupling of the piezoelectric materials due to the vibrations. In addition, due to the large rigidity of epoxy resin itself, the 1–3 type composite is stable and produces strong acoustic waves when it vibrates. However, in the type 1–3 structure after joining the silicone polymer, although the material has a certain flexibility and decoupling effect, the Young’s modulus of the piezoelectric vibrator will be reduced and the transducer acoustic transmission intensity will probably decrease. Therefore, the size and volume fraction of the piezoelectric phase of the composite and the rigidity of the whole 1–3 piezoelectric composite should be considered to improve the intensity of the transmitting acoustic signal.

#### 5.3.2. Horizontal Directivity Beam Testing of the Two Array Transducers

**Figure 17:**
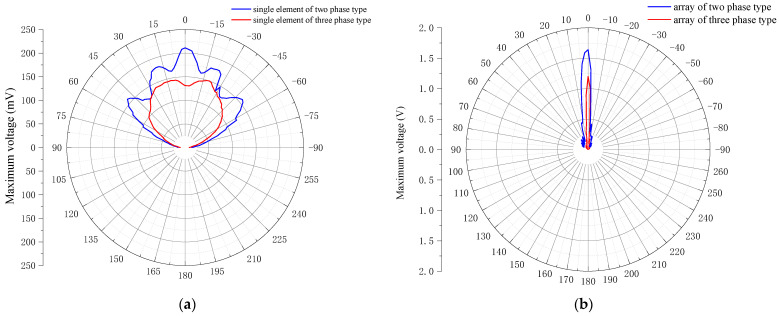
Horizontal beam directivity test curves of two transducers. (**a**) Horizontal directivity curves of single element of two types of transducers; (**b**) Horizontal directivity curves of two transducer array elements.

How large a beam can be generated by the two types of array transducers when they are independently transmitted by a single element or full array elements must be identified, which will determine the beam radiation range of the transducer acoustic field. Typically, the directivity of the array is used to read the beam width. To measure the beam directivity of the transmitting type, two array transducers were set to continuously transmit acoustic signals to the hydrophone when they are at the resonant point. Then, the metal rod with the motion controller was rotated to form an angle between the transmitter and the receiver in the horizontal direction. Every time the metal rod was rotated by 1°, the voltage peak of the oscilloscope was recorded once, and the actual horizontal directional curve can finally be obtained in this way, as shown in Figure 17.

Figure 17a presents the horizontal directivity of the two array transducers under single-element-driven transmission. Taking the −3 dB beam width, the beam width of the two-phase 1–3 type is 54°, whereas the beam width of the three-phase 1–3 type is 91°, and the acoustic radiation breadth is improved by 40.6%, indicating that the three-phase 1–3 array has a large acoustic coverage when transmitting. Moreover, the decoupling effect of flexible silicone rubber is highly obvious. When the array element of the two-phase 1–3 transducer transmits sound waves, many side lobes exist, and the acoustic energy is greatly dispersed in the main direction, whereas the array element of the three-phase 1–3 transducer has almost no side lobes, the width of the main lobe is large, and the acoustic energy is more concentrated.

Figure 17b shows the horizontal directivity of the two array transducers under the full array element-driven transmission. When all the array elements act as the driving phase, the acoustic waves generated by each array element will form a coherent superposition. Similarly, taking the −3 dB wave width of the beam transmitted by the two transducers, the beam width of the two-phase 1–3 beam is 4°, whereas the beam width of the three-phase 1–3 beam is only 3.5°. Contrastingly, the beam width of the three-phase 1–3 full array is reduced by 12.5%. Among the planar transducer arrays, the three-phase 1–3 transducer array has a more concentrated acoustic radiation beam and better directional characteristics.

## 6. Discussion

### 6.1. Experimental Error Analysis

In summary, the finite element simulation data are basically the same as those for the actual underwater tests of the transducers. However, differences exist between the simulations and the measurements. The main reasons for these differences are as follows:

1. The first is the idealization of simulation conditions. To observe the transmission performance of the transducer and simplify the calculation, only two kinds of sensitive elements were simulated here in the finite element analysis. The actual influence of the metal base, foam backing and waterproof layer of the transducer on the acoustic transmission were ignored.

2. Second, the experiment requires great accuracy. The blade thickness of the cutting machine is fixed at 0.56 mm, whereas cutting the 1-mm-wide polymer joints requires multiple piecing processes, and thus systematic errors may have been introduced when preparing the materials. The cutting pitch and kerf will directly affect the polymer width of piezoelectric composites, further influencing the volume fraction of the piezoelectric phase. Volume fractions that are too low or too high can reduce the mechanical and electrical conversion capabilities of the material, ultimately reducing the transmission performance of the transducer. Besides, the size of the piezoelectric composite determines the acoustic radiation surface of the transducer. Under the same conditions, different sizes can result in various acoustic radiation ranges and acoustic transmission intensities, which will lead to differences in transmission performance.

3. Finally, the systematic error introduced by flexible silicone rubber. Silicone rubber is a kind of flexible polymer with a low Young’s modulus, that plays a great part in the structure of three-phase 1–3 piezoelectric composites. However, silicone rubber is prone to aging and deformation when under strong voltages for a long time [26,31], and the electrode will also be damaged, which will also lead to physical differences.

In conclusion, a piezoelectric composite with a 1–3 structure for integration into the preparation of array transducer technology is proposed in this study, which has the advantages of a small volume, easy disassembly and convenient application, and the design of the array transducer’s element can allow for independent drive control, eradicating the disadvantages of the traditional transducer array design method.

### 6.2. Novelty and Significance

As the core component of underwater high-frequency sonar, the fabrication of the transducer array and the improvement of the transmission performance have always been a research issue of special concern to us. Most transducer arrays are made by hand-splicing multiple transducers to ensure that each transducer has a similar transmission performance. The transmission effect of the whole array must also be considered, which will make the production of the transducer array expensive and difficult. In this paper, the array transducer designed based on the 1–3 connected structure can not only reduce the preparation cycle of the array but also improve the overall transmission performance. However, we should also pay attention to the applicable conditions of this study. On the whole, the planar array transducer is suitable for high-frequency underwater sonar. Because of the limitations of its high-frequency work, the two array transducers are only applied to the working environment of short-range detection. Compared with the traditional hand-spliced transducer array, there are two major innovations in our design:

The mask method, which can divide a monolithic composite material into multiple elements and use multiple active materials as working elements to replace the splicing effect of multiple transducers. At the same time, the uniformly prepared 1–3 piezoelectric composite has a good consistency. It solves the problems of the long preparation cycle of traditional spliced transducer arrays, the low yield and the poor consistency of transducer array elements.

The improvement of the transmission performance of the transducer. We prepared array transducers with two composite materials with 1–3 connected structures: two-phase 1–3 composites are currently common active materials used in transducers. This active material has a high hardness and a relatively low impedance, so the transducer made of this active material has a large sound wave transmission intensity, which is suitable for the short-distance target positioning and short-distance measurement of the underwater sonar. The three-phase 1–3 type transducer has a good beam selectivity. This active material is different from the common two-phase 1–3 active materials. The addition of flexible silicone rubber can effectively reduce the coupling between the two sides of the element. Therefore, the main lobe beam width of the transmitting beam of the single element is really pure and large, so that the sound energy is maintained while working. In the full-array mode, the beam width of the three-phase transducer is smaller than that of the two-phase 1–3 transducer, which shows that the three-phase 1–3 transducer has a better orientation than the two-phase type. However, the sound transmission intensity of three-phase 1–3 transducer is slightly lower than that of the two-phase type, so this type of transducer is more suitable for underwater sonar application for target detection and target perception.

This study can provide an effective reference for the preparation and performance research of the current transducer array. In future designs, we plan to use the idea of integrating composite materials to form an array to improve the spliced transducer array.

## 7. Conclusions

In this study, two planar array transducers were designed on the basis of 1–3 connected piezoelectric composites. First, the FEA method was used to compare two kinds of composite in a water field and analyze the performance of the two composite arrays in this simulated water field. Two kinds of 1–3 piezoelectric composite array structures were fabricated using “dice-and-fill” and mask technologies. Then, the array transducer was fabricated, which improved the forming process for the transducer array and greatly reduced the fabrication period and the forming difficulty. Finally, the performance of the two transducers was measured through an underwater acoustic testing system. The experimental results show that the two 1–3 planar array transducers have good array frequency consistency and that the transmission voltage response is higher than that of a traditional transducer splicing array. The maximum transmitting voltage response of the two-phase 1–3 full array element driver can reach 179 dB, which has a strong acoustic transmission effect. It is suitable for short-range target positioning and measurement. When comparing the horizontal directivity curves of the single element and the full array of the two kinds of transducer, the three-phase 1–3 transducer has strong decoupling characteristics, which intensifies the purity of the transmitting beam. The single element of the three-phase 1–3 type transducer −3 dB acoustic beam width is up to 91°, which has a large radiation breadth and the characteristics of the whole array element acoustic transmission energy concentration. Moreover, the −3 dB beam width is 3.5°; therefore, the three-phase 1–3 type transducer has a good beam selectivity, which is suitable for short-range target detection and perception. In practical applications, the transmitting voltage response and beam width of the two transducers must be chosen, and the selection of array transducers and the working mode of array elements must be weighed. In conclusion, the array transducer composed of a 1–3 piezoelectric composite not only greatly shortens the product preparation period, but also has a large transmitting voltage response, which retains and optimizes the advantages of the traditional splicing transducer array. This new transducer array will have exceptional future application value in underwater acoustic detection, weapon guidance and ocean exploration, promoting the development of underwater ultrasonic transducers.

## Figures and Tables

**Table 1:** Parameters of each component phase in piezoelectric composites.

Parameter	PZT-5A	Epoxy	Silicon Rubber
*ρ* (kg/m^3^)	7750	1050	1000
*c*^E^_11_ (10^10^ N/m^2^)	12.1	0.36	0.004
*c*^E^_12_ (10^10^ N/m^2^)	7.54	0.138	0.0023
*c*^E^_13_ (10^10^ N/m^2^)	7.52	/	/
*c*^E^_33_ (10^10^ N/m^2^)	11.1	/	/
*s*^E^_11_ (10^−12^ m^2^/N)	16.4	278	4 × 10^5^
*s*^E^_12_ (10^−12^ m^2^/N)	−5.74	−97	2.3 × 10^5^
*s*^E^_13_ (10^−12^ m^2^/N)	−7.22	/	/
*s*^E^_33_ (10^−12^ m^2^/N)	18.8	/	/
*e*_31_ (C/m^2^)	−5.4	/	/
*e*_33_ (C/m^2^)	15.8	/	/
*ε*^S^_33_/*ε*_0_	830	4	3.3
*ε*^T^_33_/*ε*_0_	1700	4	3.3

**Table 2:** Performance test results of the two transducers in water.

	Type of Transducer	Resonant Frequency (kHz)	Anti-Resonance Frequency (kHz)	–3 dB Bandwidth (kHz)
Single array element of transducer	Two-phase 1–3 type	294.50	351.50	20.97
	Three-phase 1–3 type	269.00	359.75	21.88
Full array element of transducer	Two-phase 1–3 type	300.50	376.50	18.16
	Three-phase 1–3 type	270.75	378.50	25.52

**Table 3:** Actual test environment of the transducer.

Ambient Room Temperature	Cable Length	Depth in Water	Water Temperature	Insulation Resistance	Test Distance
21 °C	4.0 m	0.3 m	18 °C	500 MΩ	1.07 m

## Data Availability

The data presented in this study are available on request from the corresponding author.
